# Development of a UHPLC-MS method to avoid the in-source dissociation interference in characterization of crocins from Buddlejae flos and its dyeing yellow rice

**DOI:** 10.3389/fpls.2025.1659907

**Published:** 2025-10-15

**Authors:** Si Cheng, Jianing Mi, Shanshan Jiang, Arong Li, Zishao Zhong, Zhixia Chen

**Affiliations:** ^1^ The Second Affiliated Hospital of Guangzhou University of Chinese Medicine/Guangdong Provincial Hospital of Chinese Medicine, Guangzhou, China; ^2^ Chinese Medicine Guangdong Laboratory, Guangdong-Macao In-Depth Cooperation Zone in Hengqin, Zhuhai, China; ^3^ The Second School of Clinical Medicine, Guangzhou University of Chinese Medicine, Guangzhou, China; ^4^ State Key Laboratory of Traditional Chinese Medicine Syndrome, Guangzhou, China; ^5^ Guangdong Provincial Key Laboratory of Clinical Research on Traditional Chinese Medicine Syndrome, Guangzhou, China

**Keywords:** UHPLC-MS, in-source dissociation interference, Crocins, Buddlejae flos, Yellow rice

## Abstract

Buddlejae flos, the flower bud of *Buddleja officinalis* Maxim., has been used in traditional Chinese medicine to promote eye health and as a yellow food dye for cooking rice in China. Crocins are a class of essential pharmacological ingredients and edible pigments in Buddlejae flos. However, misidentification and inaccurate quantification often occur in ultra-high-performance liquid chromatography-mass spectrometry (UHPLC-MS) analysis of crocins in complex samples due to in-source dissociation and matrix effects. To avoid these interferences, we developed a UHPLC-MS approach by optimizing the chromatographic separation. In the present work, our approach facilitated the identification of crocin isomers that are usually masked by in-source dissociation species, expanding the number of detected crocins and derivatives to 28 in Buddlejae flos. Additionally, our strategy significantly enhanced the sensitivity of the UHPLC-MS method for detecting crocins in complex samples. Moreover, we performed UHPLC fingerprint analysis of 21 batches of Buddlejae flos to evaluate the geographical regionality of crocins biosynthesis. Furthermore, comparative quantification of crocins among Buddlejae flos, Gardenia fruit, and saffron reveals significant differences in the percentage of various types of crocins. The improved approach provides an informative and reliable profile of crocins in Buddlejae flos and yellow rice, which is promising for enhancing the quality control of Buddlejae flos and for potential utilization in the synthetic biology of crocins.

## 1 Introduction

Buddlejae flos, known as Mi-Meng-Hua in Chinese, is the flower bud and inflorescence of *Buddleja officinalis* Maxim. (*B. officinalis*), which is a perennial shrub in the Loganiaceae family distributed in China, Bhutan, Myanmar, and Vietnam (Xie et al., 2019). Buddlejae flos is documented in the Pharmacopoeia of the People’s Republic of China. It is frequently utilized in clinical therapies and has demonstrated positive results in terms of treatment effectiveness, particularly in the management of various ophthalmic diseases (Jiang et al., 2019; Areesanan et al., 2024). It also has a long history of application to cook rice and prepare tea by the Miao nationality and the Zhuang ethnic group (Liu et al., 2022; Wang et al., 2024). Rice cooked with Buddlejae flos aqueous extract has a more pleasant taste (Avila Acevedo et al., 2005). Buddlejae flos tea has been observed to improve ocular blood circulation, reduce visual fatigue, and mitigate stress (Wang et al., 2024). Phytochemical studies of Buddlejae flos have revealed more than 80 chemical compounds including flavonoids, phenylethanoids, triterpenoids, monoterpenoids, and so on (Jiang et al., 2019; Xie et al., 2019; Xie et al., 2020; Huang et al., 2022). Among these, a few chemical compounds have demonstrated diverse biological activities, such as anti-dry eye, anti-inflammatory, anti-oxidative, anti-diabetic, anti-obesity, and osteoporosis-improving effects, as well as the treatment of skin diseases (Jiang et al., 2019; Xie et al., 2020; Huang et al., 2022). Therefore, Buddlejae flos, with its ornamental, economic, and medicinal value, has enormous potential and extensive development opportunities, warranting further exploration.

**Figure 1:**
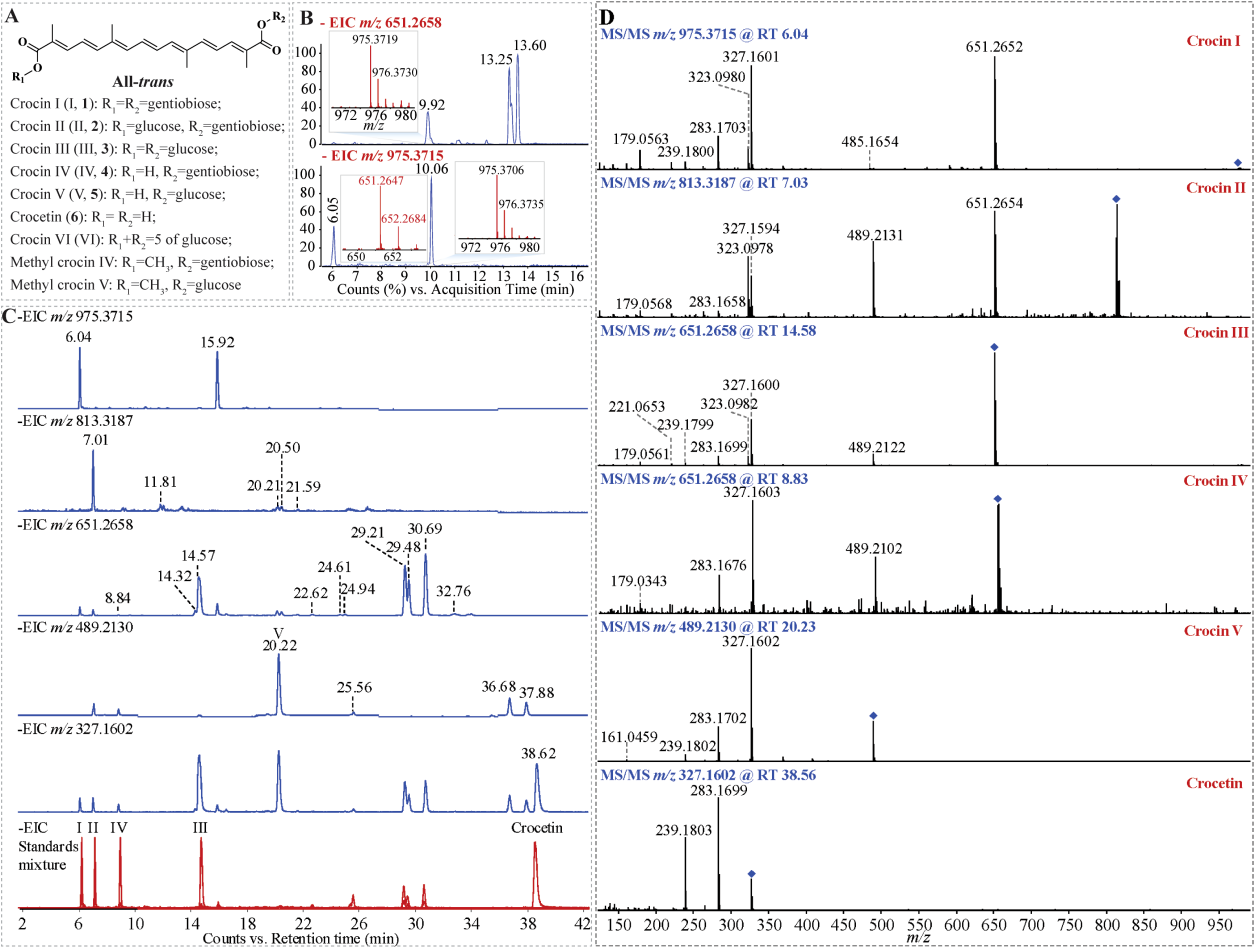
Identification of crocins and their derivatives in Buddlejae flos using UHPLC-Q-TOF-MS/MS. **(A)** The representative structures of crocins and their derivatives. **(B)** Effect of the in-source dissociation ion of crocin I isomer with an *m/z* of 975.3715 on identifying crocin III with an *m/z* of 651.2658 under weak chromatographic separation. **(C)** Elimination of the in-source dissociation interference for the identification of crocins and their derivatives by optimizing the chromatographic separation. **(D)** MS/MS spectra of crocins and their derivatives to validate their annotation.

Interestingly, crocin I, a water-soluble yellow-red pigment found in saffron and Gardenia fruit, has been recently identified in Buddlejae flos by some research groups (Huang et al., 2022). Crocins are a class of carotenoid derivatives mainly including crocin I (1), crocin II (2), crocin III (3), crocin IV (4), crocin V (5), and their *cis/trans* isomers (**Figure 1A**). Crocins and their derivatives, such as crocetin (6), possess diverse pharmacological properties, including antioxidant, anticancer, cardioprotective, neuroprotective, antidepressant, and immune-supporting activities (Asadollahi et al., 2019; Song et al., 2021; Gudarzi et al., 2022; Shahbaz et al., 2022; Tao et al., 2023). Additionally, these compounds have garnered attention due to their high economic value in the food industry, human healthcare, and cosmetics. As a natural source, saffron is also known as “red gold” due to its high value and low yield (Liu et al., 2020). Additionally, the supply of crocins from an alternative source, such as Gardenia fruit extraction, is insufficient to meet current demands (Liu et al., 2020). Therefore, there has been a surge in research into the metabolically engineered production of these valued compounds in various microorganisms and plants. For instance, tomato and tobacco have been successfully engineered as bioreactors to produce crocetin and crocins (Ahrazem et al., 2022; Zheng et al., 2022; Xie et al., 2023). Additionally, several *Escherichia coli* cell factories have been developed for the *de novo* synthesis of crocetin and crocins (Pu et al., 2020; Lee et al., 2024; Li et al., 2024). However, current strategies still suffer from insufficient yield for commercial-scale applications. Thus, this work aims to comprehensively profile crocins from Buddlejae flos, seeking to discover and extend the natural resource of crocins, which is both urgent and necessary.

Ultra-high-performance liquid chromatography-mass spectrometry (UHPLC-MS) analytical methods are the primary approach for analyzing crocins in samples with a complex matrix. However, many crocins and isomers were often co-eluted on a UHPLC column under weak chromatographic separation conditions, resulting in a significant matrix effect. In addition, crocins with more sugar moieties may undergo in-source dissociation during MS, forming lower glycosylated species that complicate accurate structure identification and quantification. To avoid the in-source dissociation interference and reduce the matrix effects, we developed a UHPLC-MS analytical approach by optimizing the chromatographic separation, which enabled the identification of crocins that are typically masked by in-source dissociation species, and enhanced the quantification of crocins in complex samples by reducing matrix effects. Additionally, based on the optimal chromatographic separation, we performed UHPLC fingerprint analysis of 21 batches of Buddlejae flos to evaluate the geographical regionality of crocins biosynthesis. Furthermore, the validated UHPLC-MS method was employed to measure crocins in yellow rice dyed with Buddlejae flos, as well as to characterize the crocins present in Buddlejae flos, Gardenia fruit, and saffron. This developed approach is a promising method for enhancing the quality control of Buddlejae flos, extending the natural resource of crocins, and promoting their potential utilization in synthetic biology.

## 2 Materials and methods

### 2.1 Materials and chemicals

LC-MS grade methanol (MeOH), acetonitrile (ACN), and formic acid (FA) were purchased from Fisher Scientific UK Ltd. (Loughborough, United Kingdom). Ultrapure water (H_2_O) was purified using a Research Scientific Instrument Co., Ltd. (China) ultrapure water system. All-*trans*-crocin I (1, catalog number: DX0011) and all-*trans*-crocin II (2, catalog number: DX0012) were purchased from Chengdu DeSiTe Biological Technology Co., Ltd. (Purity ≥ 98%, Chengdu, China). All-*trans*-crocin III (3) was obtained from TargetMol Chemicals Inc. (Catalog number: TN7052, Purity ≥ 99%, Boston, MA, United States). All-*trans*-crocin IV (4) was purchased from Shanghai Taopu Biotechnology Co., Ltd. (Catalog number: 4302, Purity ≥ 95%, Shanghai, China). All-*trans*-crocetin (6) was bought from Chengdu Purifa Technology Development Co., Ltd. (Catalog number: BP0405-100mg, Purity ≥ 98%, Chengdu, China). Japonica glutinous rice was purchased from Chaihuodayuan Rice Ltd. (Liaoning, China). Saffron and Gardenia fruit were purchased from Anhui Shenghaitang Traditional Chinese Medicine Decoction Co., Ltd. (Anhui, China) and Zhangshu Qingren Traditional Chinese Medicine Decoction Co., Ltd. (Jiangxi, China), respectively. And 21 batches of Buddlejae flos were obtained from different pharmacies (**Supplementary Table S1**). All batches of Buddlejae flos products were verified based on their Product Qualification Certificates from the companies.

### 2.2 Sample preparation

A standards mixture solution including 1, 2, 3, 4, and 6 (1.5 μg/mL each standard) was prepared in MeOH for quantitative method validation and quantitation of crocins and their derivatives.

Saffron, Gardenia fruit, and Buddlejae flos were lyophilized, powdered, and stored at −20°C within one month. Approximately 20 mg of herb powder (n = 5) was transferred into the brown glass bottles and extracted with 2 mL of MeOH in an ultrasound bath with a frequency of 40 kHz (Branson 3510 ultrasonic bath) for three times (20 min each time), followed by centrifugation for 10 min at 5,000 rpm at 4°C. The three-times supernatants (approximately 5.5 mL) were collected and combined, followed by drying with N_2_. For qualitative analysis, the residue was redissolved in 200 μL of MeOH and filtered through a 0.2 μm filter to create the sample solution for further UHPLC-MS analysis and UHPLC fingerprint analysis.

16 g of Buddlejae flos was weighed and transferred to a cooker with 0.7 L of water, and heated for 10 min. Then, 380 g of rice was added to the cooker and cooked for 15 min. After cooling, 10 g of yellow rice (n = 3) was weighed and extracted with MeOH as mentioned in the protocol above (**Supplementary Figure S1**). The supernatant was dried with N_2_ and then redissolved in 200 μL of MeOH. Then, a 0.2 μm filter was used for filtration before UHPLC-MS analysis.

### 2.3 UHPLC-UV analysis

The chromatographic separation of crocins and their derivatives was performed on an Agilent 1290 UHPLC system (Santa Clara, CA, USA) with an ACQUITY UPLC BEH C_18_ Column (Waters, 100 × 2.1 mm, 1.7 μm) and an ACQUITY UPLC BEH C_18_ Pre-Column (Waters, 5 × 2.1 mm, 1.7 μm). The column temperature was maintained at 40°C. A mixture of H_2_O/formic acid (100:0.1, v/v) (A) and ACN (B) was used as eluents at a flow rate of 0.3 mL/min. The optimized gradient program was as follows: 0–5 min, 10% B to 25% B; 5–42 min, 25% B to 41.5% B; 42–43 min, 41.5% B to 90% B, followed by washing with 90% B and equilibration with 10% B. The wavelength of crocins and their derivatives was set as 440 nm. The injection volume was 5 μL.

### 2.4 UHPLC-MS analysis

The chromatographic separation conditions for crocins and their derivatives were used as described in the protocol above. The injection volume was 5 μL. Identification of crocins and their derivatives was carried out on an Agilent UHD 6546 Q-TOF mass spectrometer (Santa Clara, CA, USA) with a Jet Stream electrospray ionization (ESI) source in the negative ion mode. Mass spectra were recorded across the range of *m/z* 100–1500; MS/MS spectra were recorded across the range of *m/z* 50–1200. The MS parameters were as follows: gas flow rate of 12 L/min, gas temperature of 350°C, sheath gas flow rate of 12 L/min, sheath gas temperature of 350°C, nebulizer of 30 psig, capillary voltage of 3.5 kV, nozzle voltage of 0.8 kV, fragmentor of 180 V, skimmer of 65, and octopoleRFPeak of 750, reference mass with 966.0007. For MS/MS measurements, different collision energies were from 10 to 40 V.

For the UHPLC-MS quantitative analysis, the sample solution should be diluted 100-fold and 500-fold with MeOH. Quantification of crocins and their derivatives was performed on an Agilent 6495 QQQ mass spectrometer (Santa Clara, CA, USA) with a Jet Stream ESI source in the negative ion mode. The MS parameters were as follows: gas flow rate of 18 L/min, gas temperature of 280°C, sheath gas flow rate of 12 L/min, sheath gas temperature of 350°C, nebulizer of 60 psig, capillary voltage of 2.5 kV, nozzle voltage of 1.0 kV, funnel high pressure RF of 150 V, funnel low pressure RF of 30 V, fragmentor of 166 V, collision cell accelerator voltage of 5 V, and collision energy of 15 V. And the tMRM transitions list was shown in **Table 1**.

**Table 1:** MRM parameters for the quantitation of 28 crocins and their derivatives in samples. CE, Collision Energy.

No.	Compound ID	RT (min)	MRM transition (*m/z*)	Fragmentor (V)	CE (V)
1	Crocin VI	5.42	1137.4→813.3	166	15
2	Crocin I	5.95	975.4→651.3	166	15
3	Crocin II	6.88	813.3→651.3	166	15
4	Crocin IV	8.69	489.2→327.2	166	15
5	Crocin VI iso-1	10.56	1137.4→813.3	166	15
6	Crocin VI iso-2	10.97	1137.4→813.3	166	15
7	Crocin II iso-1	11.67	813.3→651.3	166	15
8	Crocin VI iso-3	13.06	1137.4→813.3	166	15
9	Crocin III iso-1	14.43	651.3→327.2	166	15
10	Crocin III	14.57	651.3→327.2	166	15
11	Crocin I iso-1	15.79	975.4→651.3	166	15
12	Crocin II iso-2	20.03	813.3→651.3	166	15
13	Crocin V	20.15	489.2→327.2	166	15
14	Crocin II iso-3	20.32	813.3→651.3	166	15
15	Crocin II iso-4	21.25	813.3→651.3	166	15
16	Crocin III iso-2	22.50	651.3→327.2	166	15
17	Crocin III iso-3	24.48	651.3→327.2	166	15
18	Crocin III iso-4	24.82	651.3→327.2	166	15
19	Crocin V iso-1	25.43	489.2→327.2	166	15
20	Crocin III iso-5	29.07	651.3→327.2	166	15
21	Crocin III iso-6	29.34	651.3→327.2	166	15
22	Crocin III iso-7	30.53	651.3→327.2	166	15
23	Methyl crocin IV	32.50	711.3→323.1	166	15
24	Crocin III iso-8	32.60	651.3→327.2	166	15
25	Crocin V iso-2	36.52	489.2→327.2	166	15
26	Crocin V iso-3	37.72	489.2→327.2	166	15
27	Crocetin	38.50	327.2→239.2	166	15
28	Methyl crocin V	40.66	549.2→341.2	166	15

### 2.5 Quantitative method validation

The quantitative method was validated for crocins and their derivatives in terms of linearity, limits of detection (LOD), limits of quantification (LOQ), stability, and precision. Based on the general level of crocins in samples, the calibration linearity for crocins and crocetin was evaluated by preparing a series of standard mixture solutions at the following concentrations: 0.25, 0.5, 1, 2, 5, 10, 50, 100, 250, 500, 750, 1000, and 1500 ng/mL (three samples per concentration). Regression analysis was used to calculate linear regression equations for standards using GraphPad Prism 10 software. The LOD and LOQ were defined as the lowest concentration at which signal-to-noise ratios of approximately 3 and 10 were obtained, respectively. The stability of the analytical method was assessed through the quantitative results of three injections administered over three consecutive days, with one injection per sample per day (n = 5). Repeatability and inter-day precision of the quantitative procedure were determined based on the results of six analyses of samples within a day (n = 6) and 18 analyses of samples on three consecutive days (six samples per day, n = 18). The stability and precision were obtained by calculating the relative standard deviations (RSDs) for the levels of endogenous crocins and their derivatives in Buddlejae flos extracts. The method recovery was examined by comparing the experimental amount with the actual amount of representative standards including crocin I (240 ng or 360 ng) and crocin II (27 ng or 40 ng) spiked into samples before extraction (n = 6). The two levels were selected based on approximately 80% and 120% of the endogenous crocin I or crocin II.

### 2.6 Data analysis and statistical analysis

The high-resolution MS and MS/MS data, as well as dynamic MRM data, were processed using Agilent Mass Hunter Workstation Software. The quantitative results of crocins and their derivatives in the samples were calculated using the linear regression equations for standards. The linear regression equations of crocin III and crocetin were used for the determination of crocin VI and methyl crocins, respectively. The linear regression equations of crocins were used for the quantification of crocin derivatives. The quantitative data from the samples were converted into Microsoft Excel format and imported into GraphPad Prism 10 software (version 10.5.0) for statistical analyses. All data were presented as mean ± SD. The data were analyzed using Welch’s t-test, and p < 0.05 was considered statistically significant. A z-score, calculated using the formula z = (x - μ)/σ, where x is the value of the data point, μ is the mean of the dataset, and σ is the standard deviation, is used in data standardization to allow for comparison across different quantitative datasets from Buddlejae flos, saffron and Gardenia fruit. This approach was chosen because the data values span a wide range of magnitudes, and z-score standardization centers and scales the values, making the patterns easier to visualize in scatter plots without the influence of extreme differences in scale.

## 3 Results and discussion

### 3.1 Establishment of an improved UHPLC-MS method for the analysis of crocins and their derivatives

In MS analyses, glucose-conjugated metabolites may revert to their non-glucose-modified counterparts due to in-source collision-induced dissociation (Yan et al., 2003), a phenomenon commonly observed in the MS analysis of crocins and their derivatives. To investigate the effect of in-source dissociation interferences on the MS analysis of crocins under weak chromatographic separation conditions, the MS analysis of crocins in Buddlejae flos was performed by UHPLC-Q-TOF-MS/MS with gradient program 1 (**Figures 1B**; **S2**). The results showed that there are three prominent peaks (retention time, RT 9.92 min, 13.25 min and 13.60 min) in the extracted ion chromatography (EIC) of crocin III ion with *m/z* 651.2658 (theoretical) (**Figure 1B**). However, the MS spectrum of the ion at RT 9.92 min shows the presence of an ion with *m/z* 975, indicating the presence of crocin I or its isomer (**Figure 1B**). In addition, **Figure 1B** shows two prominent peaks (RT 6.05 min and 10.06 min) in the EIC of crocin I ion with *m/z* 975.3715 (theoretical). Similar to the MS spectrum of the ion *m/z* 651 at RT 9.92 min, molecular ions at *m/z* 975 and *m/z* 651 are both present in the MS spectrum of the ion *m/z* 975 at RT 10.06 min, indicating crocins and their isomers were co-eluted under the UHPLC gradient program 1.

**Figure 2:**
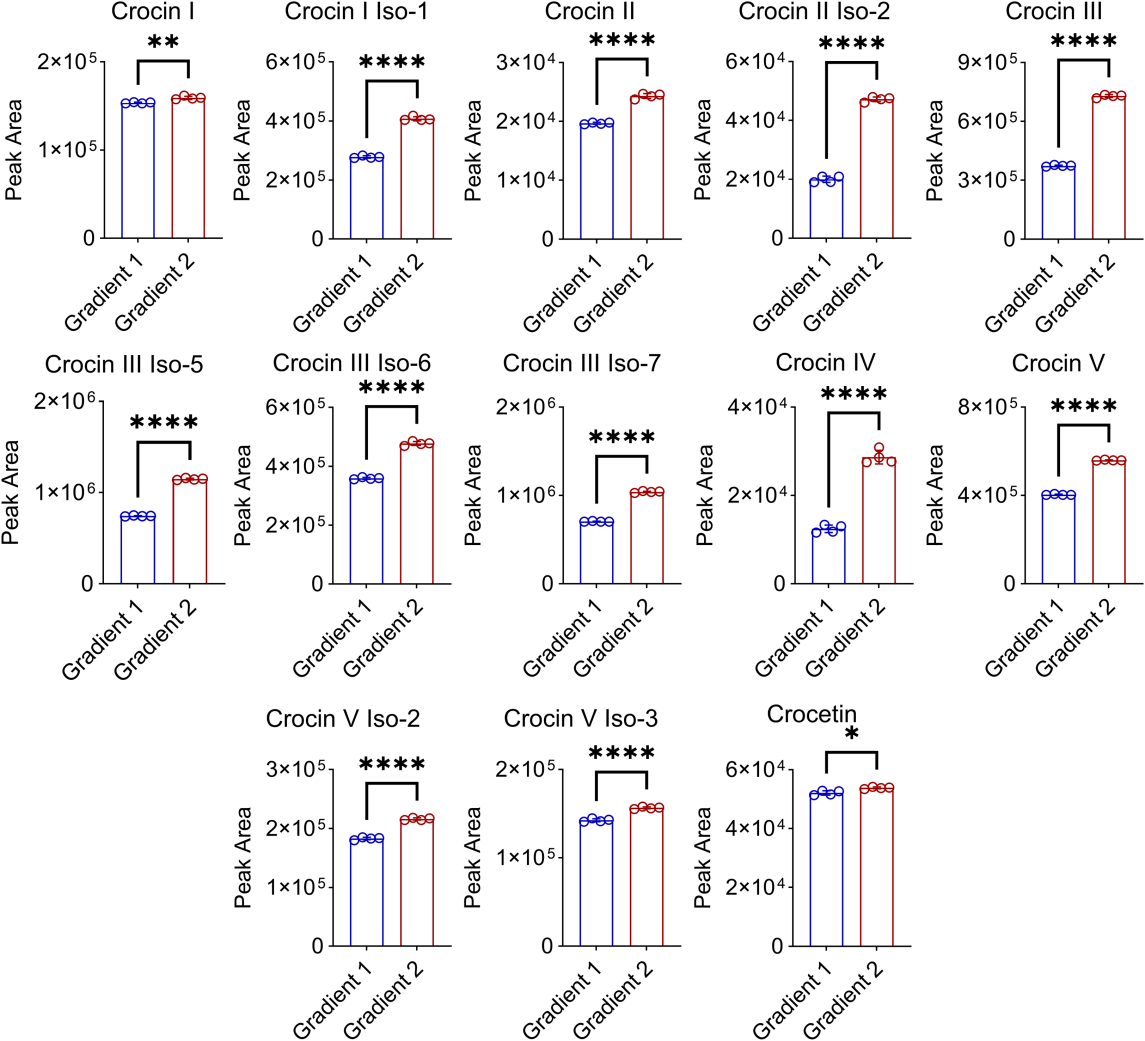
Effect of the improved chromatographic separation on the quantification of crocins in Buddlejae flos. Gradient 1: 0–5 min, 10% B to 25% B; 5–15 min, 25% B to 50% B; 15–18 min, 50% B to 95% B, followed by washing with 95% B and equilibration with 10% B. Gradient 2: 0–5 min, 10% B to 25% B; 5–42 min, 25% B to 41.5% B; 42–43 min, 41.5% B to 90% B, followed by washing with 90% B and equilibration with 10% B. All data were presented as mean ± SD, n = 4, Welch’s t-test, *p < 0.05, **p < 0.01, ****p < 0.0001.

To achieve a more suitable gradient of the mobile phases, we tested different gradient programs for the elution of crocins and their derivatives (**Supplementary Figure S2**). The results show that the finalized gradient facilitated the baseline separation of all the detected crocins and their derivatives within 40 min (**Supplementary Figure S3**). After optimizing the UHPLC gradient program, a good chromatographic separation was obtained for crocins and their derivatives (**Figure 1C**). For instance, the peaks of *m/z* 651 at RT 14.57 min and of *m/z* 975 at RT 15.92 min were separated well. As well as the two peaks of *m/z* 651 at RT 29.21 min and RT 29.48 min were separated from the overlapped peak of 651 at RT 13.25 min, as shown in **Figure 1B**. There was a *m/z* 651 ion in the MS spectra of ions *m/z* 975 at RT 6.04 min and RT 15.92 min, illuminating crocin I and crocin I isomer can produce crocin III-like ions during ionization in MS, which leads to the interference on the identification of crocin I when the co-elution occurs under UHPLC gradient program 1 (**Figure 1C**). For instance, the peaks at RT 9.92 min (**Figure 1B** upper) and at RT 10.06 min (**Figure 1B** down) show the presence of both ions with *m/z* 975 and *m/z* 651, which results in a misidentification of crocin III or crocin I isomer (**Figure 1B**). Furthermore, good chromatographic separation can reduce the ion suppression matrix effect on the detection of crocins (**Figures 2**; **S4**). **Figure 2** illuminated that our improved approach enhances the MS signal response of 13 of crocins and their derivatives including 2 of crocin Is, 2 of crocin IIs, 4 of crocin IIIs, crocin IV, 3 of crocin Vs, and crocetin, especially for crocin II iso-2, crocin III and crocin IV with an increase up to two-fold.

### 3.2 Analytical strategies for the identification of crocins and their derivatives in Buddlejae flos

The accurate mass of the targeted compound was determined by high-resolution Q-TOF-MS, providing a confident chemical formula. The feature ion from the targeted MS/MS spectrum represents the basic structure backbone of the compound candidate (**Figure 1D**, **Supplementary Figures S5-S9**). For example, an endogenous crocin II candidate (the deprotonated precursor ion at *m/z* 813.3187) co-eluted with crocin II standard at RT 7.01 min, was assigned as crocin II with the formula of C_38_H_54_O_19_ (**Figure 1C**). The MS/MS pattern of crocin II candidate shows that three of glucose group neutral ion lose, such as, from *m/z* 813 to *m/z* 651, from *m/z* 651 to *m/z* 489, and *m/z* 489 to *m/z* 327, which matched well with that of crocin II standard (**Figure 1C**; **Table 2**), confirming the assignation of endogenous crocin II extracted from Buddlejae flos material.

**Table 2:** Identification of crocins and their derivatives in Buddlejae flos extracts using UHPLC-QQQ-MS/MS.

No.	Compound ID	Chemical formula	Theoretical mass (*m/z*)*	Measured mass (*m/z*)*	RT (min)	Error (ppm)	MS/MS (*m/z*)
1	Crocin VI	C_50_H_74_O_29_	1137.4243	1137.4209	5.52	-2.99	179.0540, 221.0694, 265.1531, 323.0946, 327.1582, 383.1186, 485.1546, 651.2566, 813.3119
2	Crocin I	C_44_H_64_O_24_	975.3715	975.3716	6.04	0.10	161.0463, 179.0558, 221.0669, 239.1791, 283.1709, 323.0980, 327.1601, 341.1141, 485.1523, 489.2116, 651.2652
3	Crocin II	C_38_H_54_O_19_	813.3187	813.3180	7.01	-0.86	161.0462, 179.0560, 221.0659, 239.1790, 283.1696, 323.0964, 327.1597, 341.1126, 489.2102, 651.2666
4	Crocin IV	C_32_H_44_O_14_	651.2658	651.2660	8.84	0.31	161.0457, 179.0534, 221.0680, 239.1790, 283.1694, 323.0995, 327.1635, 489.2116
5	Crocin VI iso-1	C_50_H_74_O_29_	1137.4243	1137.4197	10.70	-4.04	179.0577, 239.1814, 327.1668, 489.2114, 813.3172
6	Crocin VI iso-2	C_50_H_74_O_29_	1137.4243	1137.4195	11.07	-4.22	161.0438, 179.0633, 221.0613, 327.1586, 485.1542, 813.3138
7	Crocin II iso-1	C_38_H_54_O_19_	813.3187	813.3182	11.81	-0.61	161.0466, 179.0570, 221.0638, 239.1791, 283.1678, 327.1607, 383.1183, 485.1507, 489.2057, 651.2676
8	Crocin VI iso-3	C_50_H_74_O_29_	1137.4243	1137.4224	13.22	-1.67	179.0594, 265.1617, 283.1684, 327.1657, 383.1126, 485.1468, 489.2109 651.2661, 813.3146
9	Crocin III iso-1	C_32_H_44_O_14_	651.2658	651.2655	14.32	-0.46	161.0462, 179.0544, 221.0649, 239.1792, 265.1577, 283.1705, 323.0987, 327.1595, 489.2110
10	Crocin III	C_32_H_44_O_14_	651.2658	651.2653	14.57	-0.77	161.0453, 179.0562, 221.0666, 239.1802, 265.1598, 283.1702, 323.0984, 327.1603, 489.2129
11	Crocin I iso-1	C_44_H_64_O_24_	975.3715	975.3709	15.92	-0.62	161.0445, 179.0551, 221.0677, 239.1799, 283.1683, 323.0956, 327.1600, 341.1101, 651.2653
12	Crocin II iso-2	C_38_H_54_O_19_	813.3187	813.3187	20.21	0.00	161.0439, 179.0591, 221.0621, 239.1803, 283.1730, 323.0927, 327.1611, 341.1108, 489.2108, 651.2672
13	Crocin V	C_26_H_34_O_9_	489.2130	489.2130	20.23	0.00	161.0459, 239.1802, 265.1600, 283.1702, 327.1602
14	Crocin II iso-3	C_38_H_54_O_19_	813.3187	813.3190	20.50	0.37	161.0436, 179.0537, 221.0692, 239.1799, 283.1694, 323.0925, 327.1617, 383.1167, 489.2106, 651.2660
15	Crocin II iso-4	C_38_H_54_O_19_	813.3187	813.3183	21.59	-0.49	161.0445, 179.0570, 221.0712, 239.1787, 265.1564, 283.1688, 323.1009, 327.1593
16	Crocin III iso-2	C_32_H_44_O_14_	651.2658	651.2657	22.62	-0.15	161.0441, 179.0569, 221.0719, 239.1764, 265.1618, 283.1713, 323.0978, 327.1575
17	Crocin III iso-3	C_32_H_44_O_14_	651.2658	651.2653	24.61	-0.77	179.0557, 221.0716, 239.1806, 265.1624, 283.1709, 327.1593
18	Crocin III iso-4	C_32_H_44_O_14_	651.2658	651.2660	24.94	0.31	161.0440, 179.0531, 221.0665, 239.1817, 283.1694, 327.1590
19	Crocin V iso-1	C_26_H_34_O_9_	489.2130	489.2130	25.56	0.00	239.1808, 265.1586, 283.1702, 327.1601
20	Crocin III iso-5	C_32_H_44_O_14_	651.2658	651.2657	29.21	-0.15	161.0487, 179.0552, 221.0679, 239.1806, 265.1607, 283.1704, 323.0963, 327.1595
21	Crocin III iso-6	C_32_H_44_O_14_	651.2658	651.2655	29.48	-0.46	161.0457, 179.0562, 221.0667, 239.1804, 265.1593, 283.1703, 323.0991, 327.1605
22	Crocin III iso-7	C_32_H_44_O_14_	651.2658	651.2657	30.69	-0.15	161.0470, 179.0561, 221.0672, 239.1802, 265.1603, 283.1705, 323.0976, 327.1602
23	Methyl crocin IV	C_33_H_46_O_14_	711.2870	711.2870	32.64	0.00	161.0459, 179.0554, 221.0676, 265.1595, 297.1906, 323.1054, 341.1733, 665.3027
24	Crocin III iso-8	C_32_H_44_O_14_	651.2658	651.2657	32.76	-0.15	161.0471, 179.0563, 221.0693, 265.1640, 283.1710, 323.0952, 327.1577
25	Crocin V iso-2	C_26_H_34_O_9_	489.2130	489.2128	36.68	-0.41	239.1805, 265.1596, 283.1698, 327.1603
26	Crocin V iso-3	C_26_H_34_O_9_	489.2130	489.2132	37.88	0.41	239.1807, 283.1709, 327.1606
27	Crocetin	C_20_H_24_O_4_	327.1602	327.1602	38.62	0.00	239.1803, 265.1594, 283.1697
28	Methyl crocin V	C_27_H_36_O_9_	549.2341	549.2336	40.79	-0.91	265.1616, 297.1917, 341.1723

* The theoretical and measured mass indicate the ions with [M+HCOO]^-^ (*m/z*) for Methyl crocin IV and Methyl crocin V, And the theoretical and measured mass indicate the ions with [M-H]^-^ (*m/z*) for others.

Due to the unavailability of crocin V and crocin isomer standards, we identified the corresponding endogenous crocins extracted from samples based on their accurate masses and high-resolution MS/MS data. For example, based on the precise mass of the deprotonated precursor ion (*m/z* 489.2130), a crocin candidate was identified as crocin V with the formula C_26_H_34_O_9_. Its MS/MS spectrum provided important fragment information for the structural elucidation (**Figure 1C**), e.g., the ions at *m/z* 327 were yielded by loss of one glucosyl group, which suggested that this crocin has one glucosyl group during the ionization; an orderly cleavage of the bonds on carbon chain gave rise to the *m/z* 283, 239 ions, which matched well with that of crocetin standard (**Figure 1C**; **Table 2**). Based on these fragment clues mentioned above, this crocin candidate was identified as crocin V.

**Figure 3:**
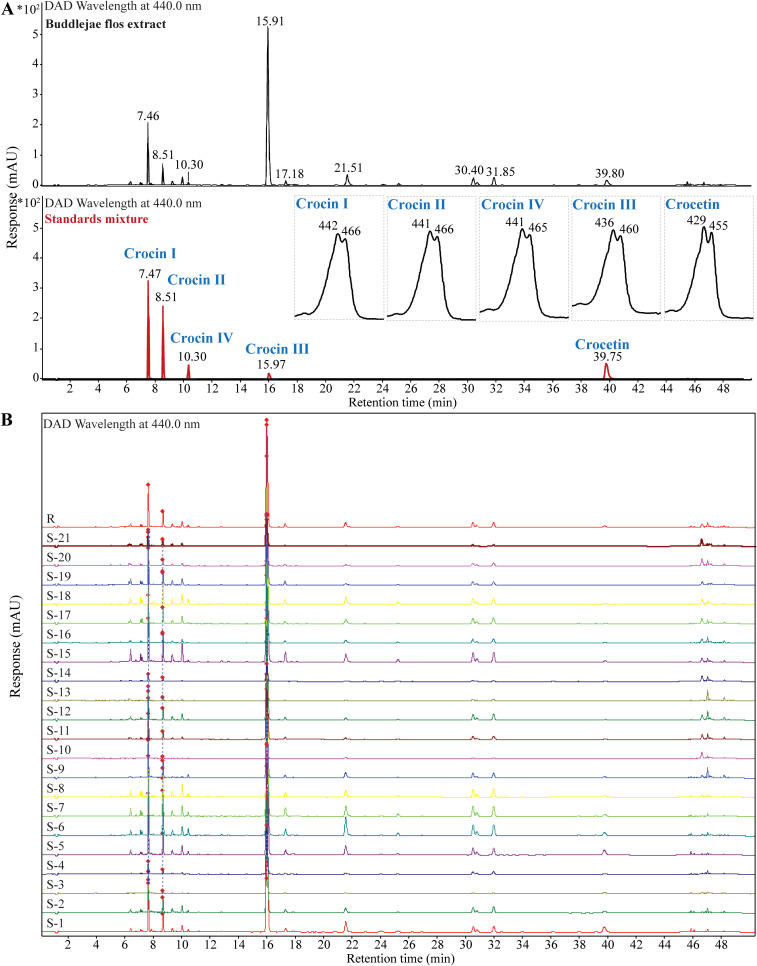
Identification of crocins and their derivatives in various batches of Buddlejae flos using UHPLC-UV. **(A)** UV chromatography of crocins and their derivatives in Buddlejae flos and a standard mixture at 440 nm wavelength. UV spectra were inserted. **(B)** UHPLC fingerprint of 21 batches of Buddlejae flos at 440 nm wavelength.

To further confirm the identification of crocins and their derivatives candidates, we obtained their characteristic UV spectra indicating that crocins and crocetin have a maximum absorption at approximately 440 nm and 460 nm using UHPLC-UV (**Figure 3A**; **Supplementary Figure S10**). In addition, UHPLC fingerprint analysis results indicate that the profile similarity of 21 batches of Buddleja flos is greater than 0.9 (**Figure 3B**).

In total, the application of this analytical strategy led to the identification of 28 endogenous crocins and their derivatives from Buddlejae flos material (**Table 2**).

### 3.3 Method validation for the quantitation of crocins and their derivatives in Buddlejae flos

**Figure 4:**
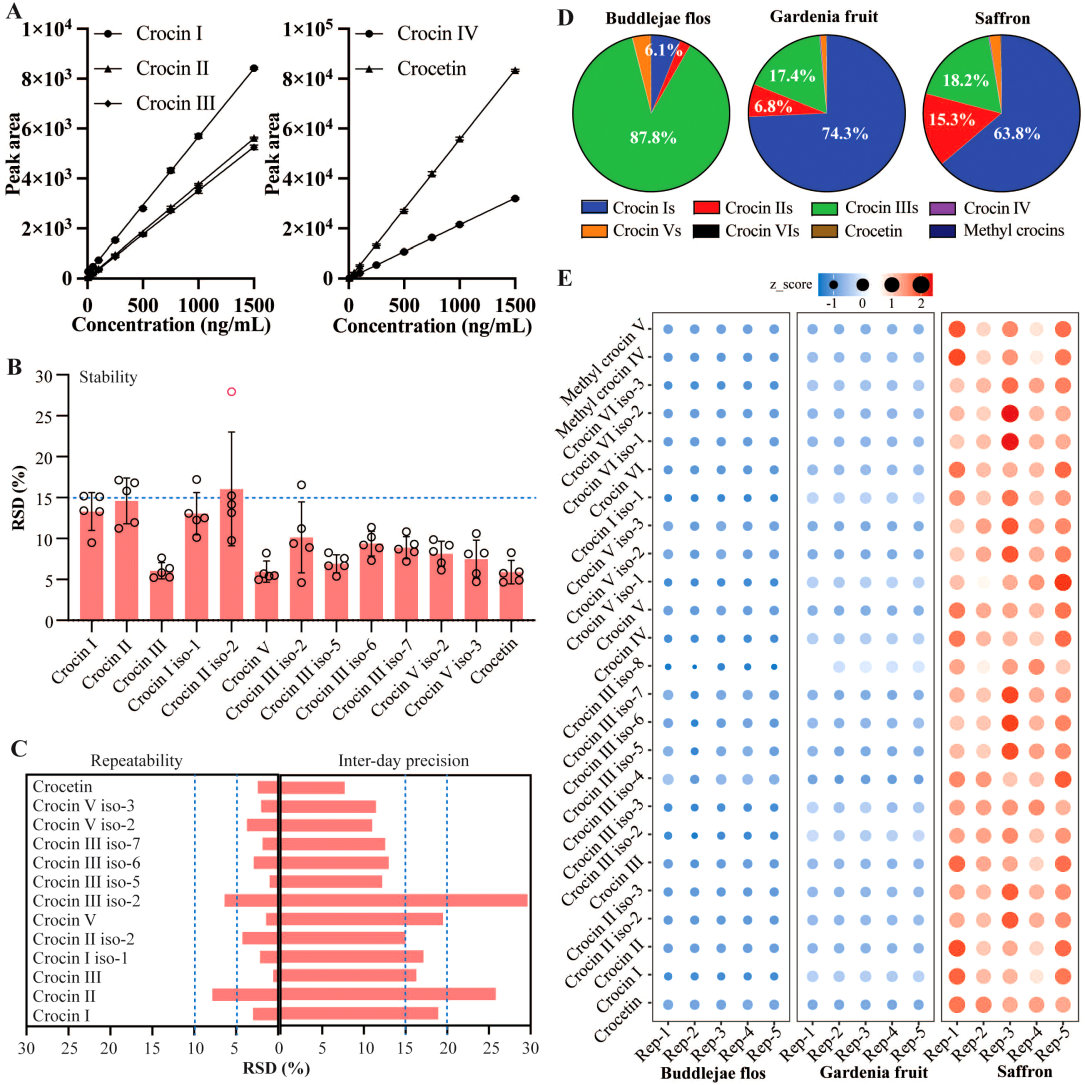
Quantitative method validation with Buddlejae flos sample and chemical standards, and quantification of crocins and their derivatives in Buddlejae flos, Gardenia fruit, and saffron. **(A)** Linearity curves for crocins and crocetin. n = 3 in each level. **(B, C)** Stability (n = 5), repeatability (n = 6), and inter-day precision (n = 18) of representative crocins and their derivatives in Buddlejae flos; Data present as Mean ± SD. **(D, E)** Percentages of different types of crocins and their derivatives, and the levels of individual crocins and their derivatives among Buddlejae flos, Gardenia fruit, and saffron (n = 5), z-score was calculated as the formular: z = (x - μ)/σ, where x is the value of the data point, μ is the mean of the dataset, and σ is the standard deviation.

As shown in **Table 2**, the calibrations reported acceptable validation parameters for quantifying crocins and their derivatives in Buddlejae flos. For crocins and crocetin standards, coefficients of determination (r^2^) higher than 0.999 were obtained, indicating high linearity within the concentration range expected for each compound in the Buddlejae flos samples. We evaluated the calibration sensitivity of crocins and crocetin through the slope of the regression line. The effect varied depending on the molecular structure of each compound. Crocin II and crocin III exhibited the lowest calibration sensitivity compared to the others, with crocetin being the most sensitive compound using the MS detector (**Figure 4A**). LOD and LOQ for crocins and their derivatives detected by MS range from 0.25 to 2 ng/mL and range from 2 to 5 ng/mL in Buddlejae flos materials, respectively (**Table 3**). Consequently, the proposed method exhibits significant sensitivity in detecting crocins and their derivatives in Buddlejae flos. In addition, the stability results of Buddlejae flos extract sample showed that RSD values were lower than 15% for all compounds quantified except for crocin II iso-2 (RSD% = 16.04%) (**Figure 4B**). Repeatability results for crocins and their derivatives of a real, lyophilized, ground Buddlejae flos sample showed RSD values lower than 5% for all compounds quantified, except for crocin II (RSD% = 7.87%) and crocin III iso-2 (RSD% = 6.41%). Inter-day precision (reproducibility) results showed that RSD values for the quantitation of crocins and their derivatives were higher than those of repeatability but always lower than 15% for more than 50% of endogenous crocins and their derivatives as well as less than 20% for 4 of low-concentration crocins (**Figure 4C**), demonstrating that the precision of our analytical approach is acceptable for the quantitation of crocins and their derivatives. The method recovery of crocin I and crocin II at two levels were [87.33% and 72.27%], and [117.49% and 92.13%], respectively (**Supplementary Table S2**).

**Table 3:** Calibration equations, correlation coefficients (r^2^), linear ranges, limit of detection (LOD), and limit of quantitation (LOQ) of crocins and crocetin standards.

Compound ID	Calibration equations	r^2^	Linear ranges (ng/mL)	LOD (ng/mL)	LOQ (ng/mL)
Crocin I	Y = 5.473X+192.6	0.9994	5-1500	0.5	2
Crocin II	Y =3.721X+27.33	0.9995	5-1500	0.5	2
Crocin III	Y =3.500X-15.46	0.9991	5-1500	1	2
Crocin IV	Y =21.36X+74.11	0.9996	5-1500	0.25	2
Crocetin	Y =55.92X-307.9	0.9997	10-1500	2	5

### 3.4 Quantitative profiling of crocins and their derivatives in Buddlejae flos, Gardenia fruit, and saffron

The validated method was applied to Buddlejae flos, Gardenia fruit, and saffron samples. The quantification of crocins and their derivatives in Buddlejae flos, Gardenia fruit, and saffron (**Figures 4D, E**) unraveled several interesting points. (1) The percentages of crocins with different numbers of sugar groups and their derivatives among Buddlejae flos, Gardenia fruit, and saffron varied greatly, with crocin IIIs (87.8%) being the most abundant crocins in Buddlejae flos; crocin Is (74.3%), followed by crocin IIIs (17.4%) in Gardenia fruit; crocin Is (63.8%), followed by crocin IIIs (18.2%) and crocin IIs (15.3%) in saffron. (2) The contents of crocins and their derivatives in saffron were significantly higher than those in Buddlejae flos and Gardenia fruit. Different patterns of crocins indicate the specificity of genes (such as UDP-glucosyltransferases, UGTs) for the biosynthesis of different crocins among Buddlejae flos, Gardenia fruit, and saffron. Different UGTs can catalyze the biosynthesis of different types of crocins from crocetin. Our previous research found that saffron CsUGT74AD1 can catalyze the formation of crocin IV and V from crocetin, while the *Nicotiana benthamiana* UGTs are primarily involved in the biosynthesis of crocin II and its isomers (Demurtas et al., 2018; Zheng et al., 2022). Additionally, the co-action of Gardenia GjUGT74F8 and GjUGT94E13 can result in the production of five types of crocins, with GjUGT94E13 being able to *in vitro* catalyze the conversion of crocin II or crocin IV to crocin I (Pu et al., 2020).

### 3.5 Quantitative profiling of crocins and their derivatives in yellow rice

**Figure 5:**
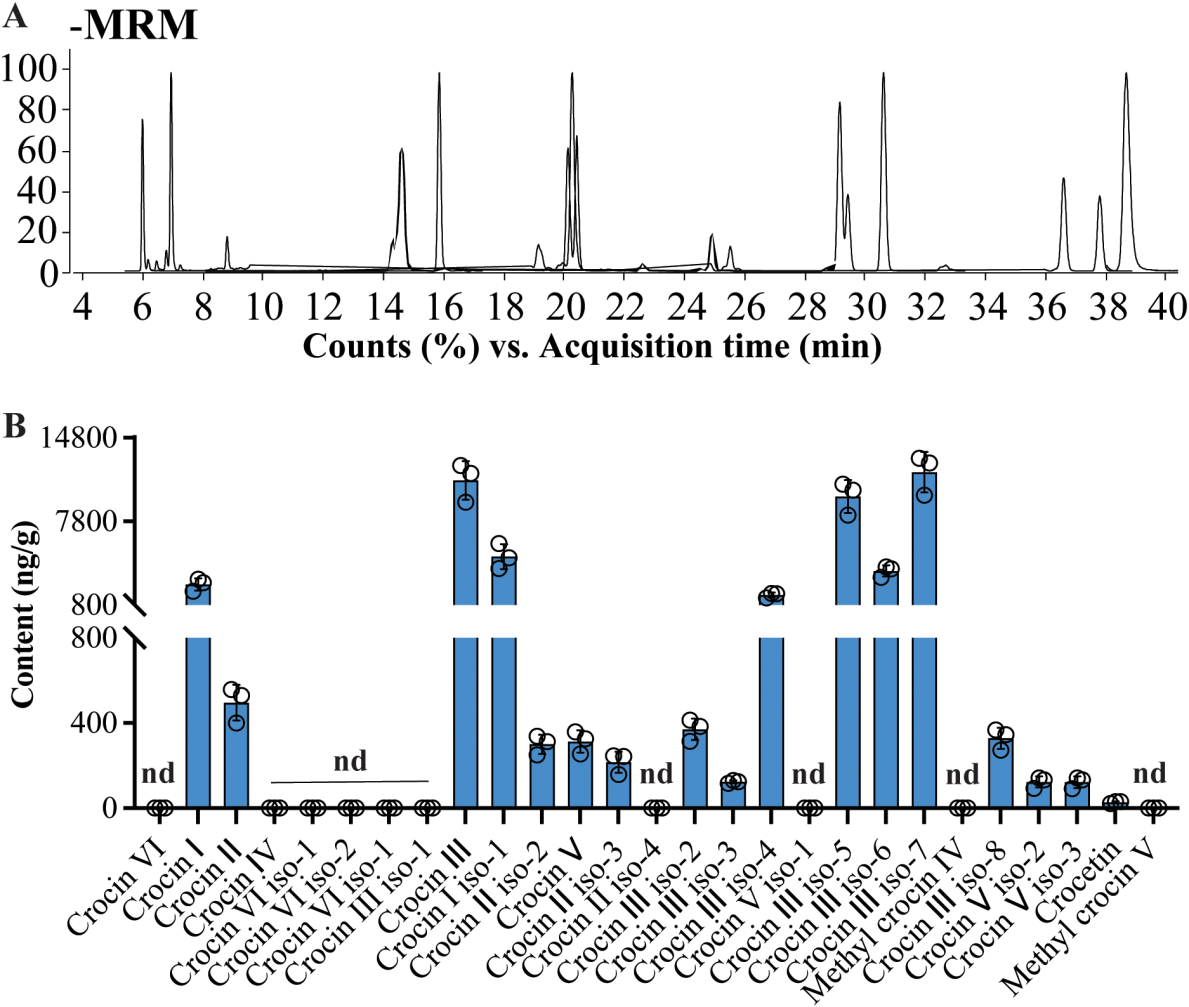
Quantitative results of crocins and their derivatives in yellow rice dyed with Buddlejae flos using UHPLC-QQQ-MS/MS. **(A)** The overlapped MRM chromatography of crocins and their derivatives detected in yellow rice extracts; **(B)** Contents of crocins and their derivatives in yellow rice extracts (nd, not detectable).

The validated method was employed to profile crocins and their derivatives in yellow rice. A total of 17 crocins and their derivatives were measured from yellow rice (**Figure 5A**). The results show that levels of all analyzed crocins and their derivatives were lower in yellow rice than in Buddlejae flos (**Figure 5B**; **Supplementary Tables S3**, **S4**). In addition, Crocin IIIs are the most abundant crocins in yellow rice (up to 41 ng/mg fresh weight), accounting for more than 80% of all crocins and their derivatives in yellow rice (**Supplementary Table S4**). Quantification of crocins and their derivatives in yellow rice samples (**Figure 5**) revealed a profile similar to that of Buddlejae flos. However, yellow rice generally contained lower levels of crocins, and their derivatives compared with Buddlejae flos.

## 4 Conclusion

In this work, we were able to characterize crocins and their derivatives using high-resolution Q-TOF MS, with the support of optimized chromatographic separation that eliminated interference from in-source dissociation species. In our new approach, we enhanced UHPLC chromatographic separation to achieve baseline separation of all detectable crocins and their derivatives within 40 min, which significantly increases their MS response. This validated approach facilitated accurate quantification of 25 crocins and their derivatives in a single run. Finally, we showed that our method is very suitable to analyze the impact of UGTs from different sources (such as Buddlejae flos, Gardenia fruit, and saffron) on their crocins and their derivatives profile, which in turn could provide crucial clues for understanding the substrate specificity of key enzymes involved in crocins biosynthesis and metabolism. The application of our method to the quantitative profiling of crocins and their derivatives from yellow rice food provided a solid foundation for further research on the functions of crocins and their derivatives as nutritional components and edible pigments in yellow rice. Additionally, this developed approach is a promising method for extending the natural resources of high-efficacy enzymes involved in the biosynthesis of crocins and promoting their potential utilization in synthetic biology.

## Data Availability

The original contributions presented in the study are included in the article/**Supplementary Material**. Further inquiries can be directed to the corresponding author.
