# Supplementary material for: Development of a UHPLC-MS method to avoid the in-source dissociation interference in characterization of crocins from Buddlejae flos and its dyeing yellow rice
Source: Front Plant Sci. 2025 Oct 15;16:1659907. doi: 10.3389/fpls.2025.1659907 (PMC12568629; doi:10.3389/fpls.2025.1659907)
Supplement: Supplementary file 1 [file DataSheet1.pdf]

Supporting Information for

Development of a UHPLC-MS Method to Avoid the In-source Dissociation Interference in Characterization of Crocins from Buddlejae Flos and Its Dyeing Yellow Rice

Si Cheng,[1,2,3,4,#] Jianing Mi,[1,2,3,4,#] Shanshan Jiang,[1,2,3,4] Arong Li,[2] Zishao Zhong, [1,2,4,5] and Zhixia Chen[1,2,5,*]

[1]The Second Affiliated Hospital of Guangzhou University of Chinese Medicine/Guangdong Provincial Hospital of Chinese Medicine, Guangzhou, 510006, China.

[2]Chinese Medicine Guangdong Laboratory, Guangdong-Macao In-Depth Cooperation Zone in Hengqin, Zhuhai, 529031, China.

[3]The Second School of Clinical Medicine, Guangzhou University of Chinese Medicine, Guangzhou, 510405, China.

[4]State Key Laboratory of Traditional Chinese Medicine Syndrome, Guangzhou, 510006, China.

[5]Guangdong Provincial Key Laboratory of Clinical Research on Traditional Chinese Medicine Syndrome, Guangzhou, 510006, China.

[#]S.C. and J.M. contributed equally to this work.

[*]Corresponding author email: chenzx116@163.com.

Content

Figure S1. A flow chart for the extraction of crocins and their derivatives.

Figure S2. Optimization of UHPLC gradient programs for the chromatographic separation of crocins and their derivatives. Short-time chromatographic separation was performed using a pre-improvement UHPLC gradient program 1: 0-5 min, 10% B to 25% B; 5-15 min, 25% B to 50% B; 15-18 min, 50% B to 95% B, followed by washing with 95% B and equilibration with 10% B; the optimal chromatographic separation was achieved with the UHPLC gradient program described in the Method section.

Figure S3. Overlapped EICs of 28 crocins and their derivatives in Buddlejae flos.

Figure S4. Enhancement of good chromatographic separation for the quantification of crocin IIs and crocin IIIs. Pre-improvement chromatographic separation was performed with a UHPLC gradient program 1; Improvement chromatographic separation was performed with the optimized gradient program.

Figure S5. MS/MS spectra of crocin I isomers from Buddlejae flos.

Figure S6. MS/MS spectra of crocin II isomers from Buddlejae flos.

Figure S7. MS/MS spectra of crocin III isomers from Buddlejae flos.

Figure S8. MS/MS spectra of crocin V isomers from Buddlejae flos.

Figure S9. MS/MS spectra of methyl crocin IV and methyl crocin V from

Buddlejae flos.

Figure S10. UV chromatography of crocins and their derivatives from Buddlejae flos, Gardenia fruit, and saffron. 440 nm wavelength.

Table S1. The information on 21 batches of Buddlejae flos.

Table S2. The method recovery of crocin I and crocin II.

Table S3. Contents of crocins and their derivatives in Buddlejae flos, Gardenia fruit, and saffron.

Table S4. Contents of crocins and their derivatives in yellow rice.

Figure S1.

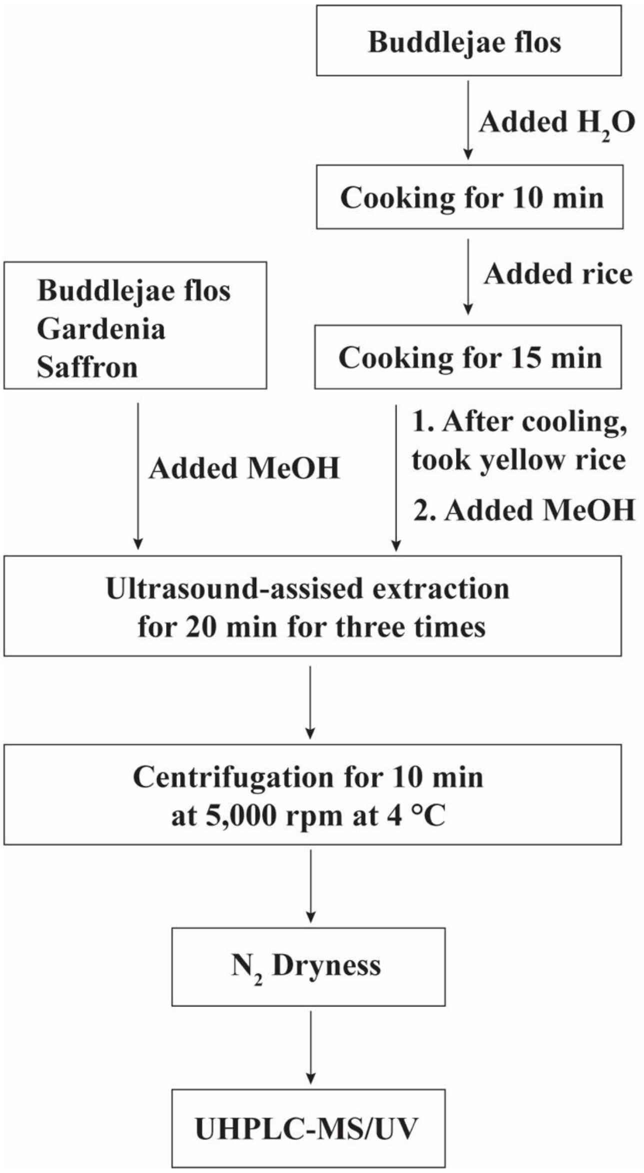
Buddlejae flos
Added H2O
Cooking for 10 min
Added rice
Cooking for 15 min
Buddlejae flos
Gardenia
Saffron
Added MeOH
1. After cooling, took yellow rice
2. Added MeOH
Ultrasound-assised extraction for 20 min for three times
Centrifugation for 10 min at 5,000 rpm at 4 °C
N2 Dryness
UHPLC-MS/UV

Figure S2.

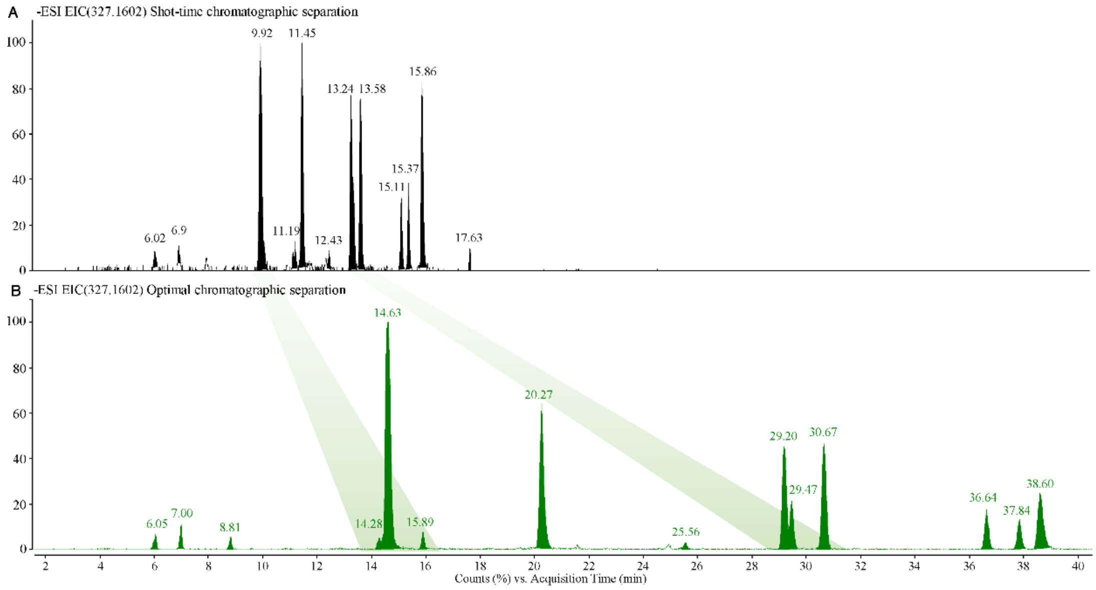

A
-ESI EIC(327.1602) Shot-time chromatographic separation
100
80
60
40
20
0
9.92
11.45
13.24
13.58
15.86
15.37
15.11
11.19
12.43
6.02
6.9
17.63
B
-ESI EIC(327.1602) Optimal chromatographic separation
14.63
20.27
29.20
30.67
29.47
36.64
37.84
38.60
6.05
7.00
8.81
14.28
15.89
25.56
2
4
6
8
10
12
14
16
18
20
22
24
26
28
30
32
34
36
38
40
Counts (%) vs. Acquisition Time (min)

Figure S3.

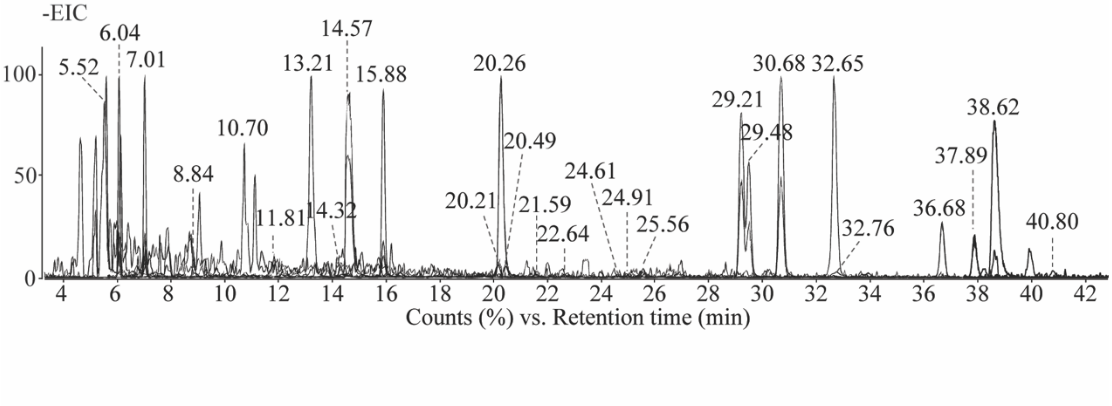
-EIC
100
50
0
5.52
6.04
7.01
8.84
10.70
11.81
13.21
14.32
14.57
15.88
20.21
20.26
20.49
21.59
22.64
24.61
24.91
25.56
29.21
29.48
30.68
32.65
32.76
36.68
37.89
38.62
40.80
4 6 8 10 12 14 16 18 20 22 24 26 28 30 32 34 36 38 40 42
Counts (%) vs. Retention time (min)

Figure S4.

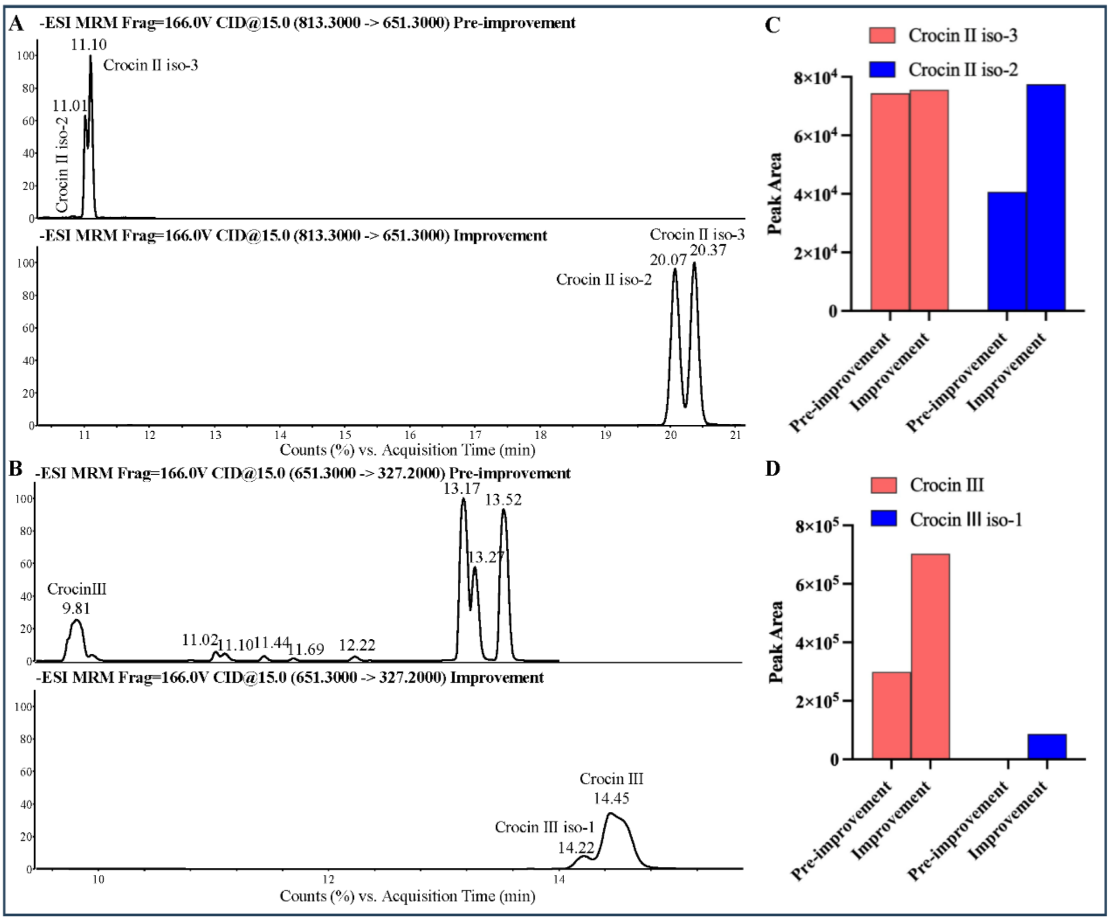

A
-ESI MRM Frag=166.0V CID@15.0 (813.3000 -> 651.3000) Pre-improvement
11.10
Crocin II iso-3
11.01
Crocin II iso-2
-ESI MRM Frag=166.0V CID@15.0 (813.3000 -> 651.3000) Improvement
Crocin II iso-3
20.07
20.37
Crocin II iso-2
Counts (%) vs. Acquisition Time (min)
B
-ESI MRM Frag=166.0V CID@15.0 (651.3000 -> 327.2000) Pre-improvement
13.17
13.52
13.27
CrocinIII
9.81
11.02
11.10
11.44
11.69
12.22
-ESI MRM Frag=166.0V CID@15.0 (651.3000 -> 327.2000) Improvement
Crocin III
14.45
Crocin III iso-1
14.22
Counts (%) vs. Acquisition Time (min)
C
Crocin II iso-3
Crocin II iso-2
Peak Area
Pre-improvement
Improvement
Pre-improvement
Improvement
D
Crocin III
Crocin III iso-1
Peak Area
Pre-improvement
Improvement
Pre-improvement
Improvement

Figure S5.

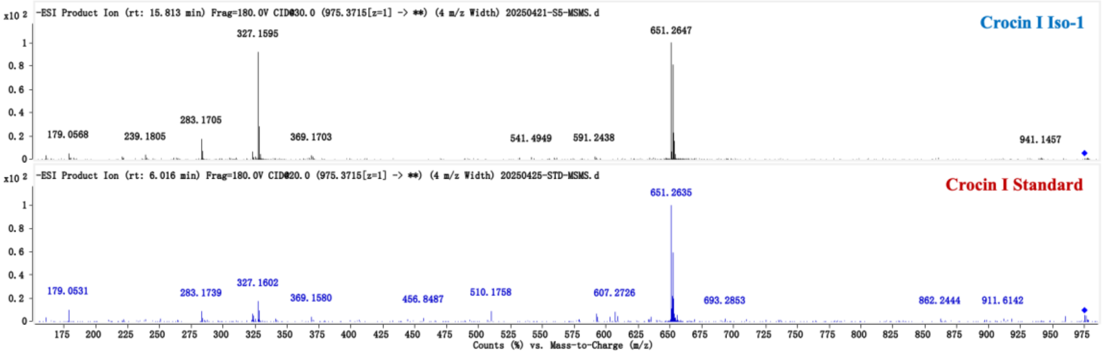
x10 2
-ESI Product Ion (rt: 15.813 min) Frag=180.0V CID@30.0 (975.3715[z=1] -> **) (4 m/z Width) 20250421-S5-MSMS.d
Crocin I Iso-1
327.1595
651.2647
283.1705
179.0568
239.1805
369.1703
541.4949
591.2438
941.1457
x10 2
-ESI Product Ion (rt: 6.016 min) Frag=180.0V CID@20.0 (975.3715[z=1] -> **) (4 m/z Width) 20250425-STD-MSMS.d
Crocin I Standard
651.2635
179.0531
283.1739
327.1602
369.1580
456.8487
510.1758
607.2726
693.2853
862.2444
911.6142
Counts (%) vs. Mass-to-Charge (m/z)

Figure S6.

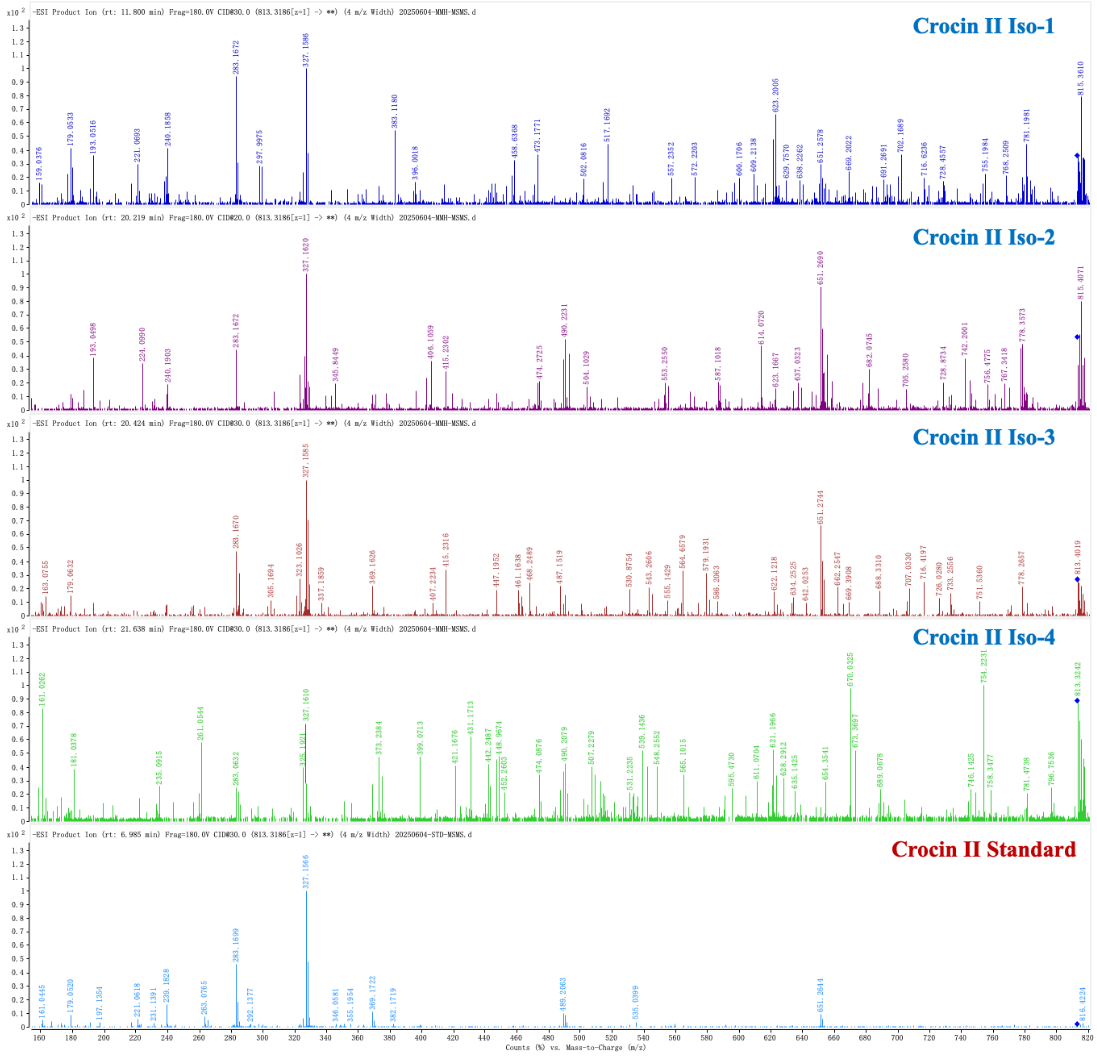
Crocin II Iso-1
Crocin II Iso-2
Crocin II Iso-3
Crocin II Iso-4
Crocin II Standard
Counts (%) vs. Mass-to-Charge (m/z)

Figure S7.

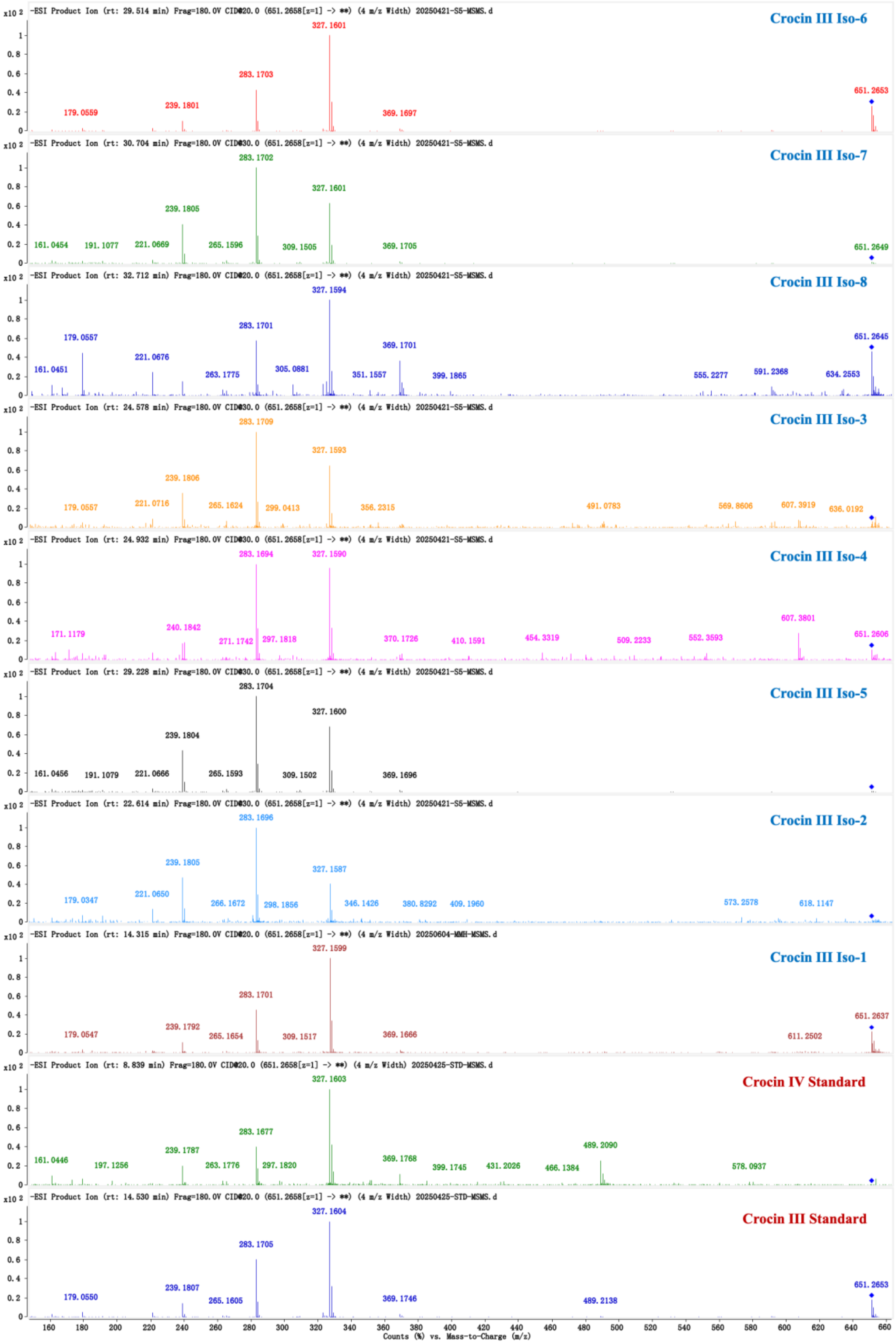
Crocin III Iso-6
Crocin III Iso-7
Crocin III Iso-8
Crocin III Iso-3
Crocin III Iso-4
Crocin III Iso-5
Crocin III Iso-2
Crocin III Iso-1
Crocin IV Standard
Crocin III Standard
Counts (%) vs. Mass-to-Charge (m/z)

Figure S8.

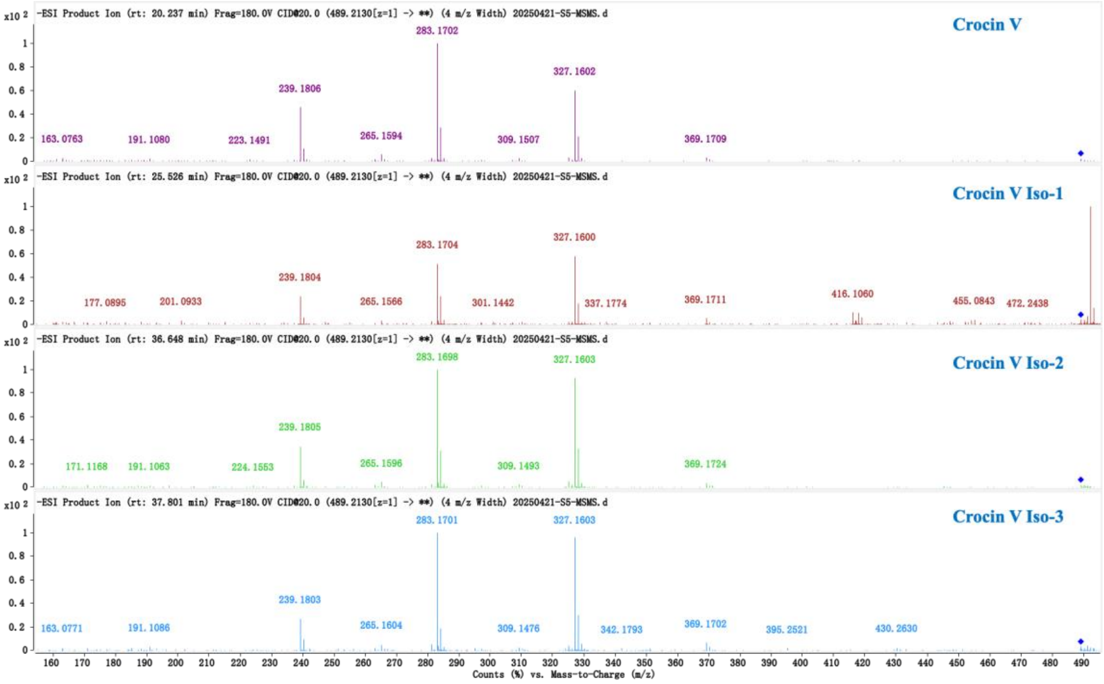

-ESI Product Ion (rt: 20.237 min) Frag=180.0V CID@20.0 (489.2130[z=1] -> **) (4 m/z Width) 20250421-S5-MSMS.d
Crocin V
163.0763
191.1080
223.1491
239.1806
265.1594
283.1702
309.1507
327.1602
369.1709
-ESI Product Ion (rt: 25.526 min) Frag=180.0V CID@20.0 (489.2130[z=1] -> **) (4 m/z Width) 20250421-S5-MSMS.d
Crocin V Iso-1
177.0895
201.0933
239.1804
265.1566
283.1704
301.1442
327.1600
337.1774
369.1711
416.1060
455.0843
472.2438
-ESI Product Ion (rt: 36.648 min) Frag=180.0V CID@20.0 (489.2130[z=1] -> **) (4 m/z Width) 20250421-S5-MSMS.d
Crocin V Iso-2
171.1168
191.1063
224.1553
239.1805
265.1596
283.1698
309.1493
327.1603
369.1724
-ESI Product Ion (rt: 37.801 min) Frag=180.0V CID@20.0 (489.2130[z=1] -> **) (4 m/z Width) 20250421-S5-MSMS.d
Crocin V Iso-3
163.0771
191.1086
239.1803
265.1604
283.1701
309.1476
327.1603
342.1793
369.1702
395.2521
430.2630
Counts (%) vs. Mass-to-Charge (m/z)

Figure S9.

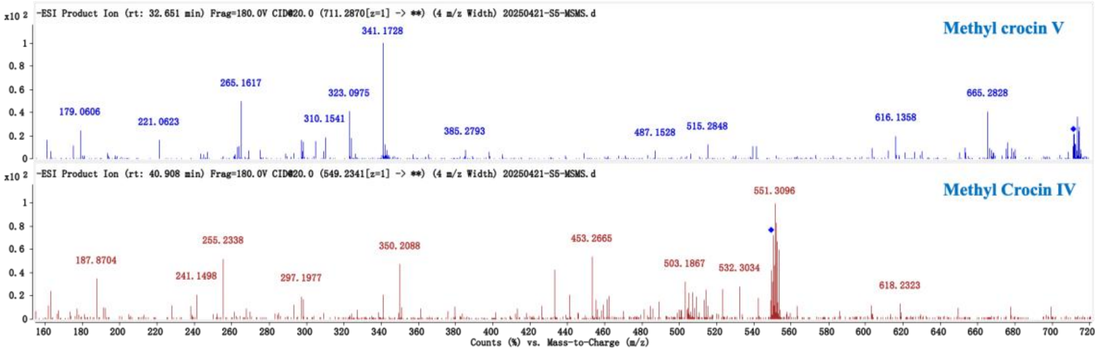
x10 2
-ESI Product Ion (rt: 32.651 min) Frag=180.0V CID@20.0 (711.2870[z=1] -> **) (4 m/z Width) 20250421-S5-MSMS.d
Methyl crocin V
341.1728
265.1617
323.0975
665.2828
179.0606
221.0623
310.1541
616.1358
385.2793
487.1528
515.2848
x10 2
-ESI Product Ion (rt: 40.908 min) Frag=180.0V CID@20.0 (549.2341[z=1] -> **) (4 m/z Width) 20250421-S5-MSMS.d
Methyl Crocin IV
551.3096
255.2338
350.2088
453.2665
187.8704
241.1498
297.1977
503.1867
532.3034
618.2323
Counts (%) vs. Mass-to-Charge (m/z)

Figure S10.

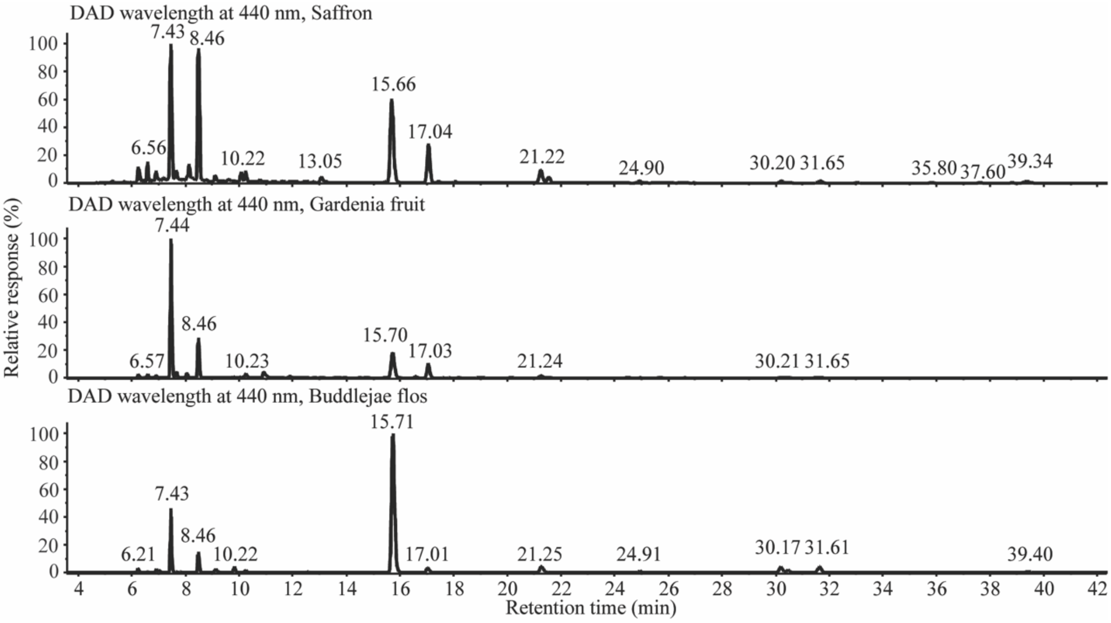
DAD wavelength at 440 nm, Saffron
7.43
8.46
15.66
17.04
6.56
10.22
13.05
21.22
24.90
30.20
31.65
35.80
37.60
39.34
DAD wavelength at 440 nm, Gardenia fruit
7.44
8.46
15.70
17.03
6.57
10.23
21.24
30.21
31.65
DAD wavelength at 440 nm, Buddlejae flos
15.71
7.43
8.46
6.21
10.22
17.01
21.25
24.91
30.17
31.61
39.40
Relative response (%)
Retention time (min)

Table S1. The information on 21 batches of Buddlejae flos.

Batch No.	Pharmacies / Companies	Date	Origin
S1	Guangzhou Baihetang Pharmacy	2017.08.16	Anhui
S2	Minshengtang Pharmacy	2019.03.01	Hubei
S3	Zhuhai Yixiantang Co., Ltd.	2019.10.08	Sichuan
S4	Renhuitang Pharmacy Baida Branch	2020.05.23	Sichuan
S5	Zhengkang Pharmacy Panyu Branch	2020.08.13	Hubei
S6	Dasenlin Lvjing Xiyuewan	2022.05.19	Sichuan
S7	Guangzhou Suihetang Co., Ltd.	2022.05.19	Sichuan
S8	Zhuhai Renzhi Health Pharmacy Co., Ltd.	2022.05.19	Sichuan
S9	Hengqin Guangji Pharmacy	2023.03.11	Sichuan
S10	Minshengtang Pharmacy	2024.04.18	Sichuan
S11	Dasenlin Pharmaceutical Group Co., Ltd.	2023.05.29	Sichuan
S12	Dasenlin Zhuhai Xiawan Zhongzhu Xincun Branch	2023.05.29	Sichuan
S13	Guangzhou Baihetang Pharmacy	2023.05.12	Sichuan
S14	Jianchuntang Lvjing Branch	2023.08.16	Anhui
S15	Rongxingtang Health Pharmacy	2023.10.23	Guangxi
S16	Rongxingtang Health Pharmacy	2023.10.23	Hubei
S17	Bozhou Jingwan Traditional Chinese Medicine Factory	2024.08.30	Sichuan
S18	Wushan Jinkang Pharmacy	2024.10.29	Sichuan
S19	Jiangxi Zhenyaotang Pharmaceutical Co., Ltd.	2024.11.16	Shandong
S20	Anhui Bozhou Guanzhong Biotechnology Co., Ltd.	2025.02.20	Sichuan
S21	Kangmei Pharmaceutical Co., Ltd.	2025.01.12	Shanxi

Table S2. The method recovery of crocin I and crocin II.

	Buddlejae flos		Buddlejae flos + 80% Standards#		Buddlejae flos + 120% Standards#	
	Crocin I (ng)&	Crocin II (ng) &	Crocin I (ng)	Crocin II (ng)	Crocin I (ng)	Crocin II (ng)
**Rep-1**	314.02	34.43	468.44	60.51	550.25	68.54
**Rep-2**	352.11	35.95	510.51	76.91	584.46	75.85
**Rep-3**	287.89	36.97	573.54	75.66	603.57	83.23
**Rep-4**	354.11	51.23	436.57	56.83	538.97	69.46
**Rep-5**	327.98	44.10	584.45	87.35	577.92	82.77
**Rep-6**	227.77	33.13	547.86	68.89	569.73	77.07
**Average**	310.65	39.30	520.23	71.02	570.82	76.15
**Recovery***	--	--	87.33%	117.49%	72.27%	92.13%

& Calibration equations of crocin I and crocin II are $Y_{(\text{crocin I})} = 17.11*X_{(\text{crocin I})} - 123.3$ and $Y_{(\text{crocin II})} = 12.03*X_{(\text{crocin II})} + 105.8$.

80% Standards: crocin I is 240 ng and crocin II is 27 ng; 120% Standards: crocin I is 360 ng and crocin II is 40 ng.

* For spiking crocin I at 240 ng, $\text{Recovery}_{(\text{Buddlejae flos + 80\% crocin I})} = [\text{Average Amount}_{(\text{Buddlejae flos + 80\% crocin I})} - \text{Average Amount}_{(\text{Buddlejae flos})}] \div 240 \times 100\%$;

For spiking crocin I at 360 ng, $\text{Recovery}_{(\text{Buddlejae flos + 120\% crocin I})} = [\text{Average Amount}_{(\text{Buddlejae flos + 120\% crocin I})} - \text{Average Amount}_{(\text{Buddlejae flos})}] \div 360 \times 100\%$;

For spiking crocin II at 27 ng, $\text{Recovery}_{(\text{Buddlejae flos + 80\% crocin II})} = [\text{Average Amount}_{(\text{Buddlejae flos + 80\% crocin II})} - \text{Average Amount}_{(\text{Buddlejae flos})}] \div 27 \times 100\%$;

For spiking crocin II at 40 ng, $\text{Recovery}_{(\text{Buddlejae flos + 120\% crocin II})} = [\text{Average Amount}_{(\text{Buddlejae flos + 120\% crocin II})} - \text{Average Amount}_{(\text{Buddlejae flos})}] \div 40 \times 100\%$.

Table S3. Contents of crocins and their derivatives in Buddlejae flos, gardenia, and saffron.

No.	Compound ID	Buddlejae flos （ug/g）					Gardenia fruit（ug/g）					Saffron（ug/g）				
		Rep-1	Rep-2	Rep-3	Rep-4	Rep-5	Rep-1	Rep-2	Rep-3	Rep-4	Rep-5	Rep-1	Rep-2	Rep-3	Rep-4	Rep-5
1	**Crocin VI**	0.23	0.12	0.30	0.52	0.37	11.11	10.13	10.92	9.98	11.39	65.81	51.62	54.36	52.77	67.85
2	**Crocin I**	193.41	68.63	120.82	227.61	184.97	15355.14	13768.10	14860.17	13918.10	15597.51	54055.51	41659.16	42764.06	35774.29	51469.06
3	**Crocin II**	45.48	17.44	33.88	38.58	29.08	1167.97	1059.76	1105.94	1081.75	1219.86	13445.62	9157.96	10178.41	8618.26	12481.70
4	**Crocin IV**	4.59	2.53	4.87	3.60	3.93	119.13	105.20	116.64	118.37	123.55	412.19	339.82	335.53	313.62	424.25
5	**Crocin VI iso-1**	0.10	0.08	0.11	0.25	0.21	7.45	7.15	7.14	6.82	8.04	47.07	48.82	77.02	51.36	52.46
6	**Crocin VI iso-2**	0.07	0.04	0.07	0.17	0.10	4.73	4.87	4.68	4.44	5.33	33.99	31.25	53.87	33.75	36.11
7	**Crocin VI iso-3**	0.53	0.27	0.64	1.08	0.81	10.36	9.74	9.88	9.15	10.70	32.67	34.10	42.90	36.77	40.85
8	**Crocin III**	1358.78	690.38	1246.16	1397.53	1157.18	2705.71	2484.42	2428.83	2410.75	2595.37	12951.85	10822.89	10839.12	9342.52	12734.14
9	**Crocin I iso-1**	264.16	104.14	164.50	300.12	256.12	18609.97	17339.29	17945.23	16920.87	19377.23	50034.96	43661.29	55867.20	43547.45	50114.24
10	**Crocin II iso-2**	68.14	30.76	51.52	57.41	53.47	1024.59	893.14	940.28	950.70	1075.85	5469.93	5112.15	6967.58	5392.88	6019.88
11	**Crocin V**	132.26	67.06	150.25	101.85	111.58	299.75	265.55	274.21	276.78	296.82	2275.93	1958.92	1990.45	1849.11	2317.22
12	**Crocin II iso-3**	68.31	28.24	51.06	58.77	58.57	936.51	814.10	851.74	915.74	953.73	5690.72	5311.69	7053.64	5385.29	6144.60
13	**Crocin III iso-2**	21.97	11.07	22.36	21.42	23.94	76.57	64.27	66.74	68.95	75.00	161.01	160.72	172.32	146.67	167.35
14	**Crocin III iso-3**	5.14	2.65	5.02	5.12	4.51	23.69	18.93	22.18	17.34	20.08	59.10	59.32	61.13	62.84	56.16
15	**Crocin III iso-4**	21.13	12.66	18.41	19.05	17.33	14.92	12.46	13.91	13.51	14.11	55.60	53.59	47.31	48.03	61.25
16	**Crocin V iso-1**	6.58	3.56	6.99	4.77	5.07	82.26	74.04	80.46	84.03	83.32	211.28	171.73	225.85	243.66	303.40
17	**Crocin III iso-5**	2317.50	1248.73	2139.51	2297.66	1909.69	2434.60	2144.96	2197.36	2212.99	2363.08	6211.36	5901.14	7952.72	6633.58	6976.78
18	**Crocin III iso-6**	634.52	352.88	586.73	632.16	540.35	799.28	742.14	770.22	763.36	845.89	1851.96	1857.29	2504.50	1945.22	2197.36
19	**Crocin III iso-7**	2006.71	1085.28	1830.02	1874.92	1683.94	2099.04	1892.88	1943.77	1862.10	2041.82	5956.88	5622.46	7567.03	6022.65	6479.97
20	**Methyl crocin IV**	0.19	0.13	0.15	0.19	0.20	4.91	4.28	4.64	4.78	4.99	31.23	20.44	25.38	18.26	27.68
21	**Crocin III iso-8**	1.62	1.09	2.32	1.96	1.43	11.75	8.99	9.99	9.24	9.54	17.95	14.29	18.26	19.32	16.32

22	**Crocin V iso-2**	68.87	38.92	77.81	53.38	57.83	87.50	78.89	77.88	80.93	88.50	463.97	522.31	657.94	510.80	593.30
23	**Crocin V iso-3**	56.30	33.41	63.48	44.38	48.91	69.78	63.84	63.63	66.41	70.28	367.67	423.72	520.57	411.68	458.23
24	**Crocetin**	13.40	11.77	11.96	13.92	12.35	14.19	13.68	13.88	12.94	14.04	102.17	97.33	87.00	90.48	87.86
25	**Methyl crocin V**	0.08	0.04	0.08	0.07	0.05	1.00	0.88	1.01	1.06	1.08	13.76	9.00	11.99	8.43	12.88

Table S4. Contents of crocins and their derivatives in yellow rice.

No.	Compound ID	Yellow rice (ng/g)		
		Rep-1	Rep-2	Rep-3
1	**Crocin I**	2664.58	2951.86	1946.78
2	**Crocin II**	556.61	529.23	399.56
3	**Crocin III**	11803.78	12480.18	9412.11
4	**Crocin I iso-1**	4743.12	5941.92	3856.10
5	**Crocin II iso-2**	313.07	337.66	249.85
6	**Crocin V**	326.20	357.71	254.57
7	**Crocin II iso-3**	242.83	246.34	158.53
8	**Crocin III iso-2**	314.06	383.34	413.04
9	**Crocin III iso-3**	129.72	124.01	116.39
10	**Crocin III iso-4**	1764.60	1749.37	1389.66
11	**Crocin III iso-5**	10424.34	10903.95	8321.26
12	**Crocin III iso-6**	3831.03	3993.45	3122.11
13	**Crocin III iso-7**	12696.01	13085.07	9980.64
14	**Crocin III iso-8**	367.62	344.78	272.46
15	**Crocin V iso-2**	133.35	142.24	93.01
16	**Crocin V iso-3**	133.43	141.07	91.09
17	**Crocetin**	29.72	30.23	21.81
